# Factors affecting the grieving process after perinatal loss

**DOI:** 10.1186/s12905-021-01457-4

**Published:** 2021-08-26

**Authors:** Makiko Kishimoto, Arisa Yamaguchi, Marina Niimura, Miki Mizumoto, Tatsuo Hikitsuchi, Kohei Ogawa, Nobuaki Ozawa, Yoshiyuki Tachibana

**Affiliations:** 1 Division of Early Childhood Mental Health, Department of Psychosocial Medicine, National Center for Child Health and Development, 2-10-1 Okura, Setagaya-ku, Tokyo, 157-8535 Japan; 2 Department of Psychosocial Medicine, National Center for Child Health and Development, Tokyo, Japan; 3 Faculty of Communication and Culture, Shoin University, Kanagawa, Japan; 4 Division of Obstetrics, Center for Maternal-Fetal, Neonatal and Reproductive Medicine, National Center for Child Health and Development, Tokyo, Japan; 5 Division of Obstetrics, Center for Maternal-Fetal, Neonatal and Reproductive Medicine, Neonatal and Reproductive Medicine, National Center for Child Health and Development, Tokyo, Japan

**Keywords:** Perinatal loss, Complicated grief, Personality trait, Developmental trait, Coping style, Attachment style

## Abstract

**Background:**

Factors associated with the grief process in response to perinatal loss have been investigated. However, few studies focused on the intrapersonal factors, such as developmental and personality traits. Hence, this study aimed to investigate medical and psychosocial risk factors, including inter- and intrapersonal factors for the development of complicated grief following perinatal loss, while considering emotional support.

**Methods:**

A total of 50 patients who were treated for grief due to perinatal loss at the National Center for Child Health and Development were divided into two groups according to the treatment period (< 6 months: n = 28; ≥ 6 months: n = 22). We compared medical and psychosocial variables between the two groups using the χ^2^ test and *t* test. All data were further analyzed using a logistic regression model to adjust for confounding effects.

**Results:**

Patients who had traits of developmental/personality disorders (adjusted odds ratio [OR]: 7.21, 95% confidence interval (CI): 1.21–42.9, *P* = .030), and those treated with psychoactive drugs (adjusted OR: 5.77, 95% CI 1.09–30.5, *P* = .039) required a longer treatment period (≥ 6 months).

**Conclusions:**

Patients with personality/developmental traits and those with active psychiatric symptoms required a more extended treatment period in response to loss, suggesting the accumulation of negative factors in these patients; thus, more intensive and specialized care is necessary for these patients. Precise analysis of the coping style, attachment style, communication skills, and life history including relationship with the original family of the patients may have implications on the approach toward patients with complicated grief after perinatal loss. Studies with larger sample size are required to increase the reliability of the present findings, and future research should address the effects of the differential attachment and coping styles of patients with developmental/personality traits on the grief process.

## Background

Perinatal loss results in a significant psychological burden on individuals, some of whom develop a long-term persistence of pathologic reactions, termed as complicated grief (CG). Grieving is not a stage-like, predictable process across time, and there are different patterns of “normal” (as well as complicated) ways of grieving [1] as well as large individual/cultural differences in reactions to loss. There are few promising risk factors of CG, which are caused in response to perinatal loss. Demographic and psychosocial factors, including older maternal age [2], a poor-quality intimate partner relationship [3, 4], lack of social support [2], couples with a history of infertility [4], and having no other living children [4], have been considered as high-risk factors for intense and prolonged grief due to perinatal loss. Pregnancy termination due to fetal anomaly can be considered as a traumatic life event with a high psychological impact [5]. Similarly, a study showed that CG developed after spontaneous abortion in almost half of the women [6]. Moreover, bereaved women had higher rates of comorbid psychiatric disorders such as depression [2, 7], anxiety disorder [2, 3], and post-traumatic stress disorder [7, 8].

Jaaniste et al. reviewed parental bereavement following the death of a child. They demonstrated that factors influencing parental bereavement outcomes consist of (1) loss-oriented stressors (e.g., circumstances surrounding the death); (2) interpersonal factors (e.g., marital factors and social support); (3) intrapersonal factors (e.g., neuroticism, trait optimism, and attachment style); and (4) coping and appraisal [9]. Among these factors, intrapersonal resources such as personality traits and the attachment style of the deceased person have attracted more attention, especially in the field of prolonged grief response to the loss of the child [10]. As interpersonal and intrapersonal factors, particularly attachment style, coping style, and personality traits, are not extensively studied among individuals who have experienced perinatal loss, evaluation of how these factors are associated with the prolonged grief response after perinatal loss is essential. However, time constraints and administrative burden are major barriers in the precise evaluation of these factors in general clinical settings. Hence, this study aimed to investigate intra- and interpersonal factors using clinical data collected during a clinical interview that are intricately related to coping and attachment styles, and personality traits. From a clinical point of view, the method that individuals use to deal with the demise of their child would be likely to correlate with how they develop relationships with others and adapt to reality. For example, individuals who experience conflicts with the family of origin, that is, the family in which they were born and raised, appear to be tormented by a sense of guilt and have difficulty in setting emotional boundaries with others and fear of forgetting about the loss. Moreover, individuals who have a conflict with their partner seem to evoke various feelings, such as anger and loneliness, due to the loss, causing difficulties in processing grief after the loss. Another factor that might prolong the grief response is having a disease [11] and being treated with psychopharmacotherapy, indicating the presence of active psychiatric symptoms. Additionally, many other key intrapersonal factors are related to the grieving process. For example, perception of self [12] and sharing experiences with others would be protective factors against CG even after the tragic loss of the child [13]. We hypothesized that patients with developmental traits who have less flexibility, lack of communication skill [14], and noneffective coping styles [15] and those with traits of personality disorder who have a higher level of nonadaptive coping styles and less mature defensive functioning [16], may develop CG after perinatal loss. However, if individuals who have experienced perinatal loss have hope for a successful subsequent pregnancy and childbirth, it may help them recover from the grief due to the perinatal loss. To provide support through a tailored approach in accordance with the needs of individuals who experienced perinatal loss, we tested our hypotheses and replicated previous findings of risk factors for CG after perinatal loss in Japanese samples.

## Methods

### Patients

This retrospective study included 50 women referred to our division for treatment of grief due to perinatal loss by the Center for Maternal–Fetal, Neonatal and Reproductive Medicine of our institution between April 2017 and March 2019. This study was approved by the institutional review board of the National Institute for Child and Health Development (Approval No.2123, Approval Date: 2/28/2020). Informed consent was obtained in the form of an opt-out option on the official website of the institution in 2019. All the study procedures were carried out in accordance with the principles in Declaration of Helsinki 1964 and its amendments later on.

### Data collection

Data on the demographic characteristics and medical information were collected from the medical records between January 2019 and April 2019. In the present study, miscarriage, stillbirth, neonatal death (i.e., death of an infant in the first 28 days of life) and abortion were all considered perinatal loss.

### Measures

Medical information, including history of physical disorder, induced abortion, fetal disease, and pharmacotherapy, was collected from the medical records. We defined “fetal disease” as a chromosomal abnormality or malformation of the fetus or neonate directly linked to the cause of death. We defined pharmacotherapy as the use of antipsychotics, mood stabilizers, antidepressants, and anxiolytics, excluding hypnotics. Psychological information, including desire for future pregnancy, conflict with the partner, and psychological conflict with the family of origin, was retrieved from the medical records, which were obtained via the therapeutic process by psychiatrists or psychologists. All patients were screened for psychiatric disorders, and diagnoses were made by a psychiatrist or medical doctor engaged in perinatal mental health care and recorded in the patients’ medical records.

Developmental disorders were defined as autism spectrum disorder (ASD) and attention deficit hyperactivity disorder (ADHD). We defined each trait as follows: autistic traits, for patients meeting the A criterion of the ASD definition; ADHD traits, for patients meeting both the A1 and A2 criteria of the ADHD definition; and personality traits, for patients meeting both the A and B criteria of personality disorder. These were assessed by a certified psychiatrist of the Japanese Society of Psychiatry and Neurology based on 5th edition of the Diagnostic and Statistical Manual of Mental Disorders (DSM-V) [17].

### Data management

Studies on comorbid personality disorders in patients with developmental disorders showed the co-occurrence of ASD/ADHD with personality disorders [18, 19]. Personality disorders from DSM-5 clusters A (odd or eccentric disorders) and C (anxious or fearful disorders) have been found frequently in patients with ASD [20], and borderline personality disorders are likely to be found in patients with ADHD [21]. As these findings suggest an overlap of clinical features between the phenotypes of ASD/ADHD and personality disorders, we classified women with psychiatric or developmental disabilities into a single category. In addition, based on several large studies that have demonstrated that gestational age (weeks) at miscarriage/stillbirth does not have a significant effect on the level of distress after perinatal loss [22], we classified women with a history of miscarriage and stillbirth into a single category.

### Statistical analyses

According to the indication that grief following miscarriage often declines significantly by 6 months [5], patients were divided into two groups according to their treatment period (< 6 months/ ≥ 6 months). The variables listed in the table were analyzed using the χ^2^ test for binomial variables and *t* test for continuous variables to assess differences in the variables of the patients between two groups with statistically significant differences (*P* < 0.05). In addition, a logistic regression analysis was performed for variables to avoid confounding effects with statistically significant differences (*P* < 0.05). The variance inflation factor was calculated by a multiple regression analysis using the same dependent and independent variables. Statistical analyses were performed using the EZR software program, version 1.5 [23, 24].

## Results

The demographic data and clinical characteristics are shown in Table 1. There were no significant differences noted between the two groups. The results of the comparison between the two groups, performed using the χ^2^ test, are presented in Table 2. Twelve patients met the A criterion of ASD, two patients met both the A1 and A2 criteria of ADHD, and one patient met both the A and B criteria of borderline personality disorder. The study showed that treatment was significantly prolonged in patients with a perceived interpersonal conflict with the original family (χ^2^ = 9.092, degrees of freedom [*df*] = 1, *P* = 0.003), developmental/personality traits (χ^2^ = 7.483, *df* = 1, *P* = 0.006), history of psychiatric disorder (χ^2^ = 9.092, *df* = 1, *P* = 0.003), and receiving psychopharmacotherapy (χ^2^ = 7.550, *df* = 1, *P* = 0.006). After the adjustment by logistic regression, developmental/personality traits (adjusted odds ratio [OR]: 7.21, 95% confidence interval [CI] 1.21–42.9, *P* = 0.030], and psychopharmacotherapy (adjusted OR: 5.77, CI 1.09–30.5, *P* = 0.039) remained significant factors (Table 3).

**Table 1:** Demographic and clinical characteristics of the patients in this study (n = 50)

Treatment period	< 6 months	≥ 6 months	*P* value
	n (%)	n (%)	
	28 (56)	22 (44)	
	Mean (SD)	Mean (SD)	
Treatment period (days)	63.18 (60.877)	679.95 (381.42)	
Age (years)	36.64 (5.38)	35.95 (5.35)	.66
Gravidity (number)	2.32 (1.83)	2.55 (1.53)	.65
Gestational age (weeks)	25.75 (10.25)	25.32 (9.90)	.88

	Yes	No	Yes	No	
	n (%)		n (%)		
Neonatal death	4 (14)	24 (86)	5 (23)	17 (77)	.44
Induced abortion	10 (36)	18 (64)	3 (14)	19 (86)	.08
Stillbirth	10 (42)	14 (58)	10 (59)	7 (41)	.28
Miscarriage	14 (58)	10 (42)	7 (41)	10 (59)	.28

*P* < .05 (*t* test) denotes statistical significance

*SD* standard deviation

**Table 2:** Comparison of factors between the two groups according to the treatment period (< 6 months vs. ≥ 6 months) using the Pearson’s χ^2^ analysis (n = 50)

Treatment period	< 6 months		≥ 6 months		*P* value
	n (%)		n (%)		
	28 (56)		22 (44)		
Factor	Yes	No	Yes	No	
	n (%)		n (%)		
History of physical disorder	10 (36)	18 (64)	10 (45)	12 (55)	.49
History of miscarriage/stillbirth	10 (36)	18 (64)	11 (50)	11 (50)	.31
History of induced abortion	2 (7)	26 (93)	4 (18)	18 (82)	.23
Fetal disease/disorder	16 (57)	12 (43)	8 (36)	14 (64)	.14
Having child/children	9 (32)	19 (68)	10 (46)	12 (54)	.34
Desire for future pregnancy	22 (96)	1 (4)	18 (90)	2 (10)	.47
Psychological conflict with partner	3 (11)	25 (89)	7 (32)	15 (68)	.06
History of psychiatric disorder	5 (18)	23 (82)	13 (59)	9 (41)	.003*
Psychological conflict with family of origin	5 (18)	23 (82)	13 (59)	9 (41)	.003*
Developmental/personality trait	4 (14)	24 (86)	11 (50)	11 (50)	.006*
Psychopharmacotherapy	7 (25)	21 (75)	14 (64)	8 (34)	.006*

EZR software program, version 1.5 was used

*P < .05 (χ^2^ test) denotes statistical significance

**Table 3:** Results of the logistic regression analysis

Variable	Odds ratio	Adjusted odds ratio	95% CI	*P* value	VIF
Psychological conflict with family of origin	6.64	3.33	0.73–15.2	.12	1.02
Developmental/personality trait	6.00	7.21	1.21–42.9	.030*	1.31
Psychopharmacotherapy	5.25	5.77	1.09–30.5	.039*	1.39
History of psychiatric disorder	6.64	1.97	0.41–9.4	.40	1.12

EZR software program, version 1.5 was used

*CI* confidence interval, *VIF* variance inflation factor

*P < .05 denotes statistical significance according to logistic regression analysis

## Discussion

The variables of the obstetric medical factor or loss-oriented stressors were not related to the grief process. As an intrapersonal factor, treatment was prolonged in patients who had received psychopharmacotherapy, and this was consistent with previous results showing an increased risk of CG in women who experienced perinatal loss, with a wide variety of psychiatric symptoms. Developmental/personality traits (even within a subclinical level) were found to be an influential risk factor for a prolonged treatment period due to grief. However, studies on the relationship of developmental/personality traits and CG after perinatal loss are limited. It is suggested that autistic traits are positively associated with a tendency to use coping styles focused on emotions and negatively associated with a tendency to cope using social diversion [15]. Adults with ADHD are assumed to have a less adaptive coping style, stronger maladaptive schemata and a sense of inadequacy that is related to lower levels of emotional well-being [25]. In addition, borderline personality disorder has been associated with increased occurrence of insecure and especially unresolved attachment representations [26]. Unresolved attachment has been linked to impaired cognitive functioning, trauma-related psychopathology, and several biological impairments related to emotional dysregulation [26]. These three types of traits recognized in our sample may represent different patterns of coping and attachment styles. However, they share common challenges in accepting help from others and sharing experience, which are essential in recovery from loss [12, 13].

Of the patients who developed CG and have developmental/personality traits (n = 11), most had perceived conflicts with the original family (n = 8). Nevertheless, of the patients who did not develop CG and have developmental/personality traits (n = 4), only one patient had perceived conflicts with the original family. This finding implied that the interrelationship of these two factors underlies the psychopathology of CG. The pervasive and severe deficits often present in children with ASD may be associated with a decreased parenting efficacy and increased parenting stress [27, 28], which indicate a risk of deteriorating child-parent relationships [28]. Children with ADHD have higher exposure to adverse childhood experience (ACE) compared with children without ADHD [29]. Specific psychosocial risks and accumulation of risk factors include conflict within family systems, strong influence on child development and behavior [29, 30]. Therefore, both development traits and conflict within family or ACE may mutually and negatively influence the intrapersonal factors. In turn, this influence negatively affects the grief process.

### Implications for practice and future research

Patients with a high risk of developing CG could be identified in clinical settings by assessing development/personality traits and preexisting/comorbid psychiatric disorders. Further investigation is warranted to explore the types and mechanisms of attachment styles and coping styles of the patients, and the factors involved in the psychopathology of CG should be further investigated. Moreover, perceived interpersonal conflict with the patient’s original family as a risk factor for developing CG, and its relationship with development/personality traits requires confirmation using a larger sample size.

The limitation of the present study, firstly, is the small sample size. Secondly, the diagnosis of developmental/personality disorders may be biased by subjective judgment because a precise psychological battery was not used. Owing to these limitations, the present study should be interpreted as a preliminary report. Nevertheless, our findings provide new insights that will aid in the further investigation of the factors affecting the relationships between psychosocial factors and perinatal loss.

## Conclusions

Our results suggested that patients with traits of developmental disorder/personality disorder, and those with active psychiatric symptoms should be carefully monitored during the grieving process. Our results should be replicated in a larger sample, and further investigation is warranted to differentiate between the types of insecure attachment and coping styles of the patients with CG after perinatal loss, as they demand a differential approach.

## Data Availability

The datasets generated during and/or analyzed during the current study are available from the corresponding author on reasonable request.

## Abbreviations

CI: Confidence interval
ACE: Adverse childhood experience
ADHD: Attention deficit hyperactivity disorder
ASD: Autism spectrum disorder
CG: Complicated grief
DSM-V: Fifth edition of the Diagnostic and Statistical Manual of Mental Disorders
OR: Odds ratio
SD: Standard deviation
VIF: Variance inflation factor
